# Associations of Diabetes and Obesity with Risk of Abdominal Aortic Aneurysm in Men

**DOI:** 10.1155/2017/3521649

**Published:** 2017-02-23

**Authors:** Lu Wang, Luc Djousse, Yiqing Song, Akintunde O. Akinkuolie, Chisa Matsumoto, JoAnn E. Manson, J. Michael Gaziano, Howard D. Sesso

**Affiliations:** ^1^Division of Preventive Medicine, Department of Medicine, Brigham and Women's Hospital, Boston, MA, USA; ^2^Division of Aging, Department of Medicine, Brigham and Women's Hospital, Boston, MA, USA; ^3^Massachusetts Veterans Epidemiology Research and Information Center and Geriatric Research, Education, and Clinical Center, VA Boston Healthcare System, Boston, MA, USA; ^4^Department of Epidemiology, Indiana University Richard M. Fairbanks School of Public Health, Indianapolis, IN, USA; ^5^Department of Clinical Epidemiology, Division of Cardiology, Tokyo Medical University Hospital, Tokyo, Japan; ^6^Department of Epidemiology, Harvard T.H. Chan School of Public Health, Boston, MA, USA

## Abstract

*Background.* The associations of diabetes and obesity with the risk of abdominal aortic aneurysm (AAA) are inconclusive in previous studies.* Subjects/Methods.* We conducted prospective analysis in the Physicians' Health Study. Among 25,554 male physicians aged ≥ 50 years who reported no AAA at baseline, 471 reported a newly diagnosed AAA during a mean of 10.4 years' follow-up.* Results.* Compared with men who had baseline body mass index (BMI) < 25 kg/m^2^, the multivariable hazard ratio (HR [95% CI]) of newly diagnosed AAA was 1.30 [1.06–1.59] for BMI 25–<30 kg/m^2^ and 1.69 [1.24–2.30] for BMI ≥ 30 kg/m^2^. The risk of diagnosed AAA was significantly higher by 6% with each unit increase in baseline BMI. This association was consistent regardless of the other known AAA risk factors and preexisting vascular diseases. Overall, baseline history of diabetes tended to be associated with a lower risk of diagnosed AAA (HR = 0.79 [0.57–1.11]); this association appeared to vary by follow-up time (HR = 1.56 and 0.63 during ≤ and >2 years' follow-up, resp.).* Conclusion.* In a large cohort of middle-aged and older men, obesity was associated with a higher risk, while history of diabetes tended to associate with a lower risk of diagnosed AAA, particularly over longer follow-up.

## 1. Introduction

Abdominal aortic aneurysm (AAA), defined as a localized dilatation of the abdominal aorta, is a significant cause of morbidity [1–3] and mortality [4–6] in aging populations. Multiple risk factors for atherosclerosis, including advanced age, male sex, smoking, hyperlipidemia, and hypertension, are associated with an increased risk of AAA [7]. However, laboratory and epidemiologic studies have suggested that the etiology of AAA may be different from the occlusive atherosclerotic disease [8].

Although individuals with diabetes have a higher risk of occlusive atherosclerotic disease, data from large-scale screening [9–13] and disease registry [14] have shown a paradoxically lower prevalence of AAA in diabetic patients. One possible explanation is that AAA may be particularly lethal in diabetic patients, and thus fewer people have both conditions at the same time. Nevertheless, other findings do not support this hypothesis. AAA progresses more slowly in diabetics [15–18] and diabetic patients are less likely to have a ruptured AAA at the time of repair [19], suggesting that diabetes or its medications may protect against the development and improve the prognosis of AAA. Meanwhile, obesity has been implicated in the pathogenesis of both diabetes and atherosclerosis, for which the key mechanisms include insulin resistance and release of adipokines. Data on the associations between measures of obesity and AAA are inconsistent; direct [20], inverse [21], and null [22] associations have all been reported. Understanding the complex associations of diabetes and obesity with AAA could provide important insight into the unique etiology of AAA and guide more effective prevention and treatment strategies.

To further elucidate the association between diabetes and obesity with the risk of AAA, we conducted a prospective analysis in a large cohort of US male physicians who reported clinical diagnosis of AAA over a mean of 10.4 years' follow-up.

## 2. Materials and Methods

### 2.1. Study Population

The Physicians' Health Study (PHS) consists of two completed randomized clinical trials. The PHS I was a randomized, double-blind, placebo-controlled 2 × 2 factorial trial testing aspirin and *β*-carotene in the primary prevention of cardiovascular disease (CVD) and cancer among 22,071 US male physicians aged 40–84 years in 1982 [23]. The PHS II was a 2 × 2 × 2 × 2 factorial trial testing *β*-carotene, vitamin E, vitamin C, and a multivitamin in the prevention of CVD, cancer, eye disease, and cognitive function among 14,641 US male physicians aged ≥ 50 years in 1996-1997, including 7,641 PHS I participants and 7,000 newly recruited physicians [24]. All interventions in PHS have ended and follow-up continues as an ongoing observational study. Both trials and posttrial observational follow-up were approved by the Brigham and Women's Hospital institutional review board. All participants provided written informed consent.

**Figure 1:**
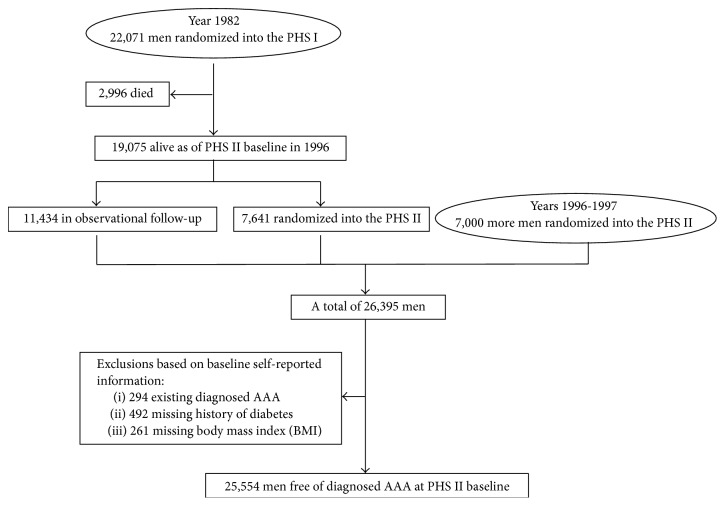
Flow diagram of participants in the Physicians' Health Study (PHS) I and II that are included in the current analysis.

For the current study, we included 19,075 PHS I participants who were alive at PHS II baseline starting in 1996, plus 7,000 newly recruited PHS II participants for analysis (Figure 1). We excluded those with missing information on baseline body mass index (BMI) (*n* = 261) or history of diabetes (*n* = 492). We also excluded 294 participants who reported a history of AAA at PHS II baseline. As a result, 25,554 men initially free of clinically diagnosed AAA were included for analyses.

### 2.2. Identification of Diagnosed Abdominal Aortic Aneurysm

PHS II participants were asked to report a clinical diagnosis (including month and year of diagnosis) of AAA on enrollment and each annual follow-up questionnaire. We used ICD-9 codes of 441.3 (ruptured AAA) and 441.4 (AAA without mention of rupture) to identify AAA. Incident cases were defined as men who were free of diagnosed AAA at baseline and reported a newly diagnosed AAA during follow-up. Through March 2012, a total of 471 newly diagnosed AAAs were reported, with 427 cases also reporting the date of diagnosis. For the 44 cases that did not report the diagnosis date, the date of event was assigned to when the annual questionnaire with reported AAA was received. Only three of these 44 cases had missed an adjacent annual follow-up before the self-report of AAA, for whom the interval between the actual and the assigned diagnosis date may be longer.

A previous study has validated self-reported AAA repair and rupture by extensive medical record review and demonstrated sensitivity and specificity of 90% and 100%, respectively [25]. In a validation study in PHS, we reviewed medical records for a subset of 77 participants who reported AAA and subsequently developed major CVD events including myocardial infarction and stroke. Clinically diagnosed AAAs were confirmed in 47 (61%) of the 77 self-reported cases, with documented imaging examinations (*n* = 15), repair procedures (*n* = 33), or rupture events (*n* = 2). Of the remaining self-reported cases, we could not confirm nor disconfirm AAA based on available records. Since this validation study used medical records that were retrieved primarily for adjudication of myocardial infarction and/or stroke, it is possible that the diagnosis of less severe CVD conditions such as untreated and unruptured small AAA may not be recorded. We anticipate a higher confirmation rate if medical records were more extensively searched for AAA diagnoses.

### 2.3. Baseline Covariates

On the PHS II baseline questionnaires, men provided self-reports of age (in years), height and weight (used to calculate BMI in kg/m^2^), cigarette smoking (never, past, and current), alcohol use (none, monthly, weekly, and daily), and vigorous exercise (none, 1–3 times/month, 1-2 times/week, 3-4 times/week, and 5–7 times/week). Diabetes (yes, no) was defined by the history of a physician diagnosis. In a previous validation study conducted in a subsample of PHS participants, diabetes was confirmed in 59 (98.3%) of 60 self-reported cases via medical record view by two independent physicians [26]. Hypertension was defined by any history of antihypertensive treatment, self-reported systolic blood pressure ≥ 140 mmHg, or diastolic blood pressure ≥ 90 mmHg. Hyperlipidemia was defined by the history of a physician diagnosis, cholesterol-lowering treatment, or self-reported total cholesterol ≥ 240 mg/dL.

### 2.4. Data Analyses

All statistical analyses were conducted using SAS (SAS Institute, Cary, NC, USA) version 9.3, with a two-sided significance level at 0.05. We first compared the baseline characteristics of men who reported newly diagnosed AAA with those who did not. The association of diagnosed AAA with known risk factors was assessed using generalized linear models for continuous variables or chi-square tests for categorical variables. Cox proportional hazards regression models estimated the hazard ratios (HRs) and 95% CIs of diagnosed AAA in association with baseline BMI and history of diabetes. Person-years of follow-up were calculated for each participant from PHS II baseline to the date of AAA diagnosis, the date of last known information, or March 2012, whichever came first. BMI was modeled first as categorical variable (normal-weight: <25 kg/m^2^, overweight: 25–<30 kg/m^2^, and obese: ≥30 kg/m^2^) and then as continuous variable with a quadratic term included to assess possible curvilinearity. History of diabetes was modeled as a binary variable. The assumption of proportional hazard was violated in the model for history of diabetes (*P* for the interaction term of diabetes with logarithm of follow-up time < 0.05). We therefore estimated the HRs for diabetic versus nondiabetic men within and beyond the first 2 years of follow-up, after which the interaction term with logarithm of follow-up time was no longer significant. We considered known risk factors for AAA including age, race, smoking status, alcohol use, vigorous exercise, history of hypertension, hypercholesterolemia, and CVD as potential confounders and also possible effect modifiers. Interactions were tested by including product-terms. We also examined the cumulative incidence curve for diagnosed AAA according to baseline BMI and history of diabetes across entire follow-up time.

## 3. Results

Among 25,554 men who reported no AAA at PHS II baseline, 471 reported a new diagnosis of AAA during a mean of 10.4 (maximum: 14.5) years' follow-up. Compared with men who remained free of diagnosed AAA, those who had newly diagnosed AAA were older, had higher BMI and greater alcohol consumption, engaged in lower levels of vigorous exercise, were more likely to be current smokers, and have a history of hypertension, hypercholesterolemia, and CVD at baseline (Table 1). The proportion of men with history of diabetes at baseline was similar in those who did and did not have new diagnosis of AAA (*P* > 0.05).

**Figure 2:**
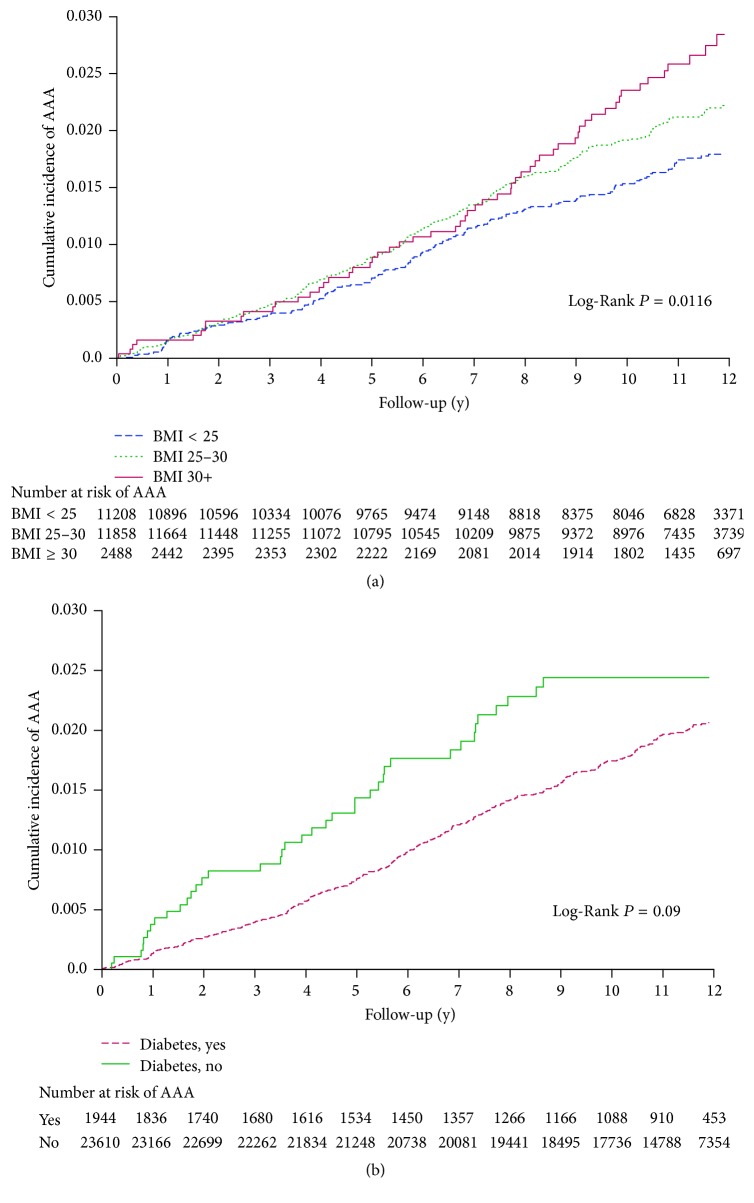
Cumulative incidence of clinically diagnosed AAA according to baseline BMI (a) and history of diabetes (b). Multivariable model adjusted for age, race, randomized treatment assignment, smoking status, alcohol use, vigorous exercise, history of cardiovascular disease, hypertension, and hypercholesterolemia. Model for BMI also adjusted for history of diabetes, and vice versa.

After adjusting for age, race, and randomized treatment (aspirin, *β*-carotene, vitamin E, vitamin C, and multivitamin), the risk of diagnosed AAA was higher with increasing BMI at baseline (Table 2). Compared with men who had normal baseline BMI, the HR of diagnosed AAA was 1.44 (95% CI: 1.18–1.75) for overweight men and 2.11 (95% CI: 1.56–2.85) for obese men. After additional adjustment for lifestyle factors (smoking, alcohol use, and vigorous exercise) and clinical factors (history of hypertension, hypercholesterolemia, CVD, and diabetes), the associations were attenuated but remained statistically significant, with corresponding HRs of 1.30 (95% CI: 1.06–1.59) and 1.69 (95% CI: 1.24–2.30), respectively. These associations were consistent across categories of known AAA risk factors (all *P* for interaction > 0.05). The multivariable HRs of AAA for obese versus normal-weight men ranged from 1.38 to 1.87 in subgroups of age, smoking status, alcohol use, and vigorous exercise. Higher BMI was also associated with increased risk of clinically diagnosed AAA regardless of the presence or absence of CVD, hypertension, and hypercholesterolemia. In stratified analysis by history of diabetes, a positive association between BMI and risk of AAA was found only in men without, but not in those with, history of diabetes, but the interaction was not statistically significant (*P* = 0.30).  Figure 2(a) showed that the cumulative incidence of AAA was higher among overweight and obese men than normal-weight men across the entire follow-up time. When BMI was modeled as a continuous variable, the risk of diagnosed AAA was 6% higher (multivariable HR: 1.06, 95% CI: 1.03–1.08) for each unit increase in baseline BMI (data not shown).

Comparing diabetic versus nondiabetic men, the overall HR of clinically diagnosed AAA was 1.08 (95% CI: 0.78–1.50) in the basic model and 0.79 (95% CI: 0.57–1.11) in the multivariable model (Table 3). The lack of an association was consistent among men stratified by baseline age, smoking status, alcohol use, vigorous exercise, history of hypertension, hypercholesterolemia, and CVD (all *P* for interaction > 0.05). The association between history of diabetes and risk of diagnosed AAA appeared to vary by follow-up time; men with baseline diabetes had a slightly higher rate of diagnosed AAA during the first 2 years of follow-up (multivariable HR: 1.56, 95% CI: 0.85–2.85), but significantly lower rate of AAA during prolonged follow-up beyond 2 years (HR: 0.63, 95% CI: 0.42–0.94). Comparing the cumulative incidence curves of AAA for men with versus without baseline history of diabetes, the two curves diverged after the first 1-2 years of follow-up (Figure 2(b)).

## 4. Discussion

In this prospective cohort of US male physicians with a mean of 10.4 years' follow-up, we found that baseline obesity was associated with a higher risk of clinically diagnosed AAA. This association remained significant after adjusting for potential confounders and was consistent across categories of known AAA risk factors. In contrast, there is suggestive evidence that history of diabetes might be associated with a lower risk of diagnosed AAA that occurred after 2 years of follow-up. Our findings of the complex associations of diabetes and obesity with risk of AAA highlight a unique pathogenesis of AAA with important clinical and public health implications.

The majority of previous epidemiologic studies on diabetes and AAA have been cross-sectional studies [9–14] and noted an inverse correlation between presence of AAA and diabetes. Prospective studies remain only a few. In 161,808 postmenopausal women from Women's Health Initiative (WHI), baseline diabetes was inversely (odds ratio: 0.29, 95% CI: 0.13–0.68) associated with AAA events that were symptomatic or required intervention and had been documented with a diagnostic or interventional procedure [27]. In 39,352 men from the Health Professionals Follow-Up Study (HPFS) [28], there was a nonstatistically significant inverse association (HR: 0.55, 95% CI: 0.26–1.17) between baseline diabetes and subsequent self-reported AAA confirmed by medical record review. In 104,813 men and women from the Kaiser Multiphasic Health Checkup Cohort Study [29], history of diabetes was also nonsignificantly associated with a lower risk of AAA identified from hospitalization discharge diagnoses and/or repair procedures (HR: 0.62, 95% CI: 0.36–1.05). Furthermore, in a study using Taiwanese insurance data, the incidence rate of thoracic and abdominal aortic aneurysm was significantly lower (HR: 0.64, 95% CI: 0.56–0.74) in 160,391 patients with diabetes compared with 646,710 subjects without diabetes [30]. Other studies [31–35], including the Tromso study that screened all participants by ultrasonography at baseline and 7-year follow-up to assess AAA [34], found no association of impaired glucose tolerance or blood glucose level with incident AAA morbidity or mortality, before or after adjustment for other risk factors. Finally, a meta-analysis that combined data from 6 prospective studies published between 1993 and 2013 found a significant lower incidence of AAA in diabetic versus nondiabetic individuals (odds ratio: 0.54, 95% CI: 0.31–0.91; *P* = 0.03) [36].

In the present study, we found a significant inverse association of diabetes history with AAA diagnosed after >2 years of follow-up. Laboratory studies have provided biological plausibility that diabetes may reduce the risk of AAA during prolonged period of time. The hyperglycemia-induced advanced glycation leads to cross-linking of elastin and collagen in the extracellular matrix within abdominal aorta wall [37]. This alteration inhibits secretion of metalloproteinases and prevents excessive proteolysis, a key process in formation and progression of AAA [17]. Hyperglycemia also modulates expression of thrombosis and fibrinolysis factors [38], which may reduce clot degradation and renewal of intraluminal thrombus in AAA and thus improve aneurysmal wall stability and slow the rate of expansion [39, 40]. Exposure to glycated and cross-linked collagen lattices also limits chronic inflammation to the intima and protect the media from matrix degradation [41]. Alternatively, lifestyle changes after diabetes diagnosis, such as smoking cessation, weight control and exercise, and medication treatment for diabetes including metformin [42] and rosiglitazone [43], may also have favorable effects against AAA development and explain the lower risk during longer-term follow-up.

Our study also found an association between greater BMI and an increased risk of AAA, consistent across varying cardiovascular risk factor profiles. Though multiple lifestyle factors correlated with obesity may explain this association, there is evidence that pathogenic processes related to obesity, such as the release of adipokines and obesity-induced aortic inflammation, may lead to vessel weakening and subsequent aneurysmal formation [44]. Previous prospective studies of obesity and AAA have obtained contradictory results. The HPFS [28] and Whitehall study [35] each reported a positive association of baseline BMI with subsequent AAA determined by review of medical records or death certificates, a finding consistent with ours. The Kaiser study [29] and two population-based Swedish cohort studies [45] found a borderline significant positive association of abdominal obesity, defined by waist circumference or Sagittal abdominal diameter, with AAA identified from hospitalization discharge diagnoses and/or death registries. However, in four other studies [27, 33, 34, 46], including two studies that performed standardized ultrasound scans to assess AAA based on aortic diameter [34, 46], measures of obesity at baseline were not associated with incident AAA during follow-up.

Strengths of our study include the prospective analysis, a large number of diagnosed AAA cases, and comprehensive information on covariates. However, an important methodologic consideration of our study is the ascertainment of AAA by self-reported clinical diagnosis on questionnaires. Since participants were not screened, majority of cases identified in our study are likely symptomatic AAA. Missing of asymptomatic and undiagnosed cases may lead to underestimation of the disease rate in our study. On the other hand, since the PHS participants are US male physicians, the self-reported disease outcomes have been fairly well validated by medical record review, with confirmation rates among records reviewed ranging from 60% to 80% for major CVD and cancers. Despite the limitation in outcome ascertainment, our study provides relevant data on risk factors for clinically manifest AAA. Our finding of an association between BMI and AAA suggests that obesity could potentially be an independent risk factor. Nevertheless, more studies are needed in women and diverse populations to confirm this finding and determine appropriate AAA screening recommendations based on obesity status. In contrast, the unclear and inconclusive relation between diabetes history and subsequent AAA argues against change in current guidelines for AAA screening and management with regard to diabetes status.

Other limitations of this study are also noted. First, although the validity of self-reported disease outcomes [47] and anthropometries [48] in health professionals is high, we cannot rule out random error in these measurements. In addition, our analyses were confined to baseline BMI and history of diabetes, with no data on the duration of exposures or changes over time. These random misclassifications would tend to bias the estimate of associations toward the null. Second, although the male physicians in PHS were less likely to overreport a diagnosis of AAA, elderly men with diabetes or obesity are subject to more intense medical care and closer surveillance, and thereafter the likelihood of finding vascular complications including AAA might be higher. Third, limited by the existing data, we cannot further examine the association with history of diabetes according to diagnosis criteria, type and duration of treatment, and presence of complications. Finally, the PHS cohort consists of predominantly white, elderly US men. Findings from this study may not be generalizable to women and other populations.

## 5. Conclusion

In this large, prospective cohort of US male physicians, we found that obesity was positively associated with risk of clinically diagnosed AAA. There is suggestive evidence that history of diabetes may be associated with a lower risk of AAA over long-term follow-up. Additional studies are warranted to confirm or refute these associations.

## Figures and Tables

**Table 1:** Baseline characteristics of men who had versus who did not have newly diagnosed abdominal aortic aneurysm (AAA) during a mean of 10.4 years' follow-up.

Baseline characteristics	Newly diagnosed AAA	No AAA	*P* ^*∗*^
*N*	471	25083	
Age, years	70.8 ± 7.4	65.4 ± 9.0^†^	<0.0001
White, %	93.2	90.5^‡^	0.11
Body mass index, kg/m^2^	26.3 ± 3.3	25.8 ± 3.4	0.001
Smoking, %			<0.0001
Never	25.5	53.6	
Former	62.4	42.8	
Current	11.9	3.6	
Alcohol use, %			0.005
Never	17.6	17.9	
Monthly	7.6	7.1	
Weekly	30.4	37.6	
Daily	42.9	35.3	
Vigorous exercise, %			0.0004
Never	44.4	35.4	
1–3 times/month	1.9	2.8	
1-2 times/week	11.9	15.9	
3-4 times/week	28.9	28.7	
>5 times/week	12.3	15.4	
Disease history, %			
Cardiovascular disease	23.1	11.2	<0.0001
Diabetes	8.5	7.6	0.46
Hypertension	63.1	45.2	<0.0001
Hypercholesterolemia	51.4	41.0	<0.0001

^*∗*^ *P* for *t*-test for continuous variables and for chi-square test for categorical variables.

^†^Mean ± SD is shown for continuous variables.

^‡^Percentages are shown for categorical variables.

**Table 2:** Hazard ratio of clinically diagnosed AAA in association with baseline body mass index (BMI) overall and in subgroups.

BMI categories	*N*	Basic model^*∗*^	*P*, interaction	Multivariable model^†^	*P*, interaction
All men					
<25 kg/m^2^	174	1.00 (ref)		1.00 (ref)	
25–<30 kg/m^2^	238	1.44 (1.18–1.75)		1.30 (1.06–1.59)	
≥30 kg/m^2^	59	2.11 (1.56–2.85)		1.69 (1.24–2.30)	
Age			0.93		0.89
<65 years					
<25 kg/m^2^	30	1.00 (ref)		1.00 (ref)	
25–<30 kg/m^2^	49	1.35 (0.86–2.13)		1.12 (0.70–1.77)	
≥30 kg/m^2^	18	2.16 (1.20–3.88)		1.59 (0.86–2.92)	
≥65 years					
<25 kg/m^2^	144	1.00 (ref)		1.00 (ref)	
25–<30 kg/m^2^	189	1.42 (1.14–1.76)		1.31 (1.05–1.63)	
≥30 kg/m^2^	41	1.95 (1.37–2.78)		1.62 (1.13–2.33)	
Smoking status			0.70		0.68
Never					
<25 kg/m^2^	51	1.00 (ref)		1.00 (ref)	
25–<30 kg/m^2^	59	1.33 (0.91–1.95)		1.30 (0.89–1.91)	
≥30 kg/m^2^	10	1.53 (0.77–3.05)		1.44 (0.71–2.90)	
Ever					
<25 kg/m^2^	123	1.00 (ref)		1.00 (ref)	
25–<30 kg/m^2^	178	1.35 (1.07–1.70)		1.25 (0.99–1.58)	
≥30 kg/m^2^	49	1.94 (1.39–2.72)		1.64 (1.16–2.32)	
Alcohol use			0.40		0.38
Less than daily					
<25 kg/m^2^	85	1.00 (ref)		1.00 (ref)	
25–<30 kg/m^2^	140	1.62 (1.24–2.13)		1.49 (1.13–1.96)	
≥30 kg/m^2^	37	2.28 (1.54–3.38)		1.84 (1.23–2.76)	
More than daily					
<25 kg/m^2^	85	1.00 (ref)		1.00 (ref)	
25–<30 kg/m^2^	95	1.30 (0.97–1.75)		1.13 (0.84–1.53)	
≥30 kg/m^2^	22	2.17 (1.35–3.50)		1.72 (1.06–2.80)	
Vigorous exercise			0.63		0.51
Less than once per week					
<25 kg/m^2^	72	1.00 (ref)		1.00 (ref)	
25–<30 kg/m^2^	109	1.33 (0.99–1.80)		1.27 (0.93–1.72)	
≥30 kg/m^2^	37	2.10 (1.40–3.15)		1.87 (1.23–2.84)	
More than once per week					
<25 kg/m^2^	101	1.00 (ref)		1.00 (ref)	
25–<30 kg/m^2^	128	1.48 (1.14–1.92)		1.32 (1.01–1.72)	
≥30 kg/m^2^	21	1.79 (1.11–2.87)		1.38 (0.85–2.24)	
Cardiovascular disease			0.71		0.68
No					
<25 kg/m^2^	131	1.0 (ref)		1.0 (ref)	
25–<30 kg/m^2^	186	1.49 (1.19–1.87)		1.35 (1.08–1.70)	
≥30 kg/m^2^	45	2.14 (1.52–3.02)		1.72 (1.21–2.45)	
Yes					
<25 kg/m^2^	43	1.0 (ref)		1.0 (ref)	
25–<30 kg/m^2^	52	1.17 (0.78–1.76)		1.11 (0.73–1.68)	
≥30 kg/m^2^	14	1.68 (0.90–3.14)		1.52 (0.80–2.87)	
Hypertension			0.21		0.25
No					
<25 kg/m^2^	73	1.0 (ref)		1.0 (ref)	
25–<30 kg/m^2^	79	1.37 (0.99–1.88)		1.27 (0.92–1.76)	
≥30 kg/m^2^	21	2.82 (1.72–4.62)		2.42 (1.46–3.99)	
Yes					
<25 kg/m^2^	101	1.0 (ref)		1.0 (ref)	
25–<30 kg/m^2^	158	1.34 (1.04–1.73)		1.28 (0.99–1.65)	
≥30 kg/m^2^	38	1.53 (1.04–2.25)		1.39 (0.94–2.06)	
Hypercholesterolemia			0.33		0.21
No					
<25 kg/m^2^	81	1.0 (ref)		1.0 (ref)	
25–<30 kg/m^2^	118	1.62 (1.22–2.15)		1.50 (1.12–2.00)	
≥30 kg/m^2^	24	1.99 (1.26–3.16)		1.59 (0.99–2.55)	
Yes					
<25 kg/m^2^	91	1.0 (ref)		1.0 (ref)	
25–<30 kg/m^2^	117	1.25 (0.95–1.64)		1.12 (0.85–1.49)	
≥30 kg/m^2^	34	2.13 (1.42–3.18)		1.75 (1.16–2.66)	
Diabetes					
No			0.27		0.30
<25 kg/m^2^	161	1.0 (ref)		1.0 (ref)	
25–<30 kg/m^2^	216	1.45 (1.18–1.78)		1.32 (1.07–1.63)	
≥30 kg/m^2^	54	2.31 (1.69–3.16)		1.84 (1.34–2.53)	
Yes					
<25 kg/m^2^	13	1.0 (ref)		1.0 (ref)	
25–<30 kg/m^2^	22	1.21 (0.60–2.42)		1.00 (0.49–2.03)	
≥30 kg/m^2^	5	0.91 (0.31–2.69)		0.69 (0.23–2.07)	

^*∗*^Model adjusted for age, race, randomized treatment assignment (aspirin, *β*-carotene, vitamin E, vitamin C, multivitamin).

^†^Model additionally adjusted for smoking status (never, past, current), alcohol use (none, monthly, weekly, daily), vigorous exercise (<1 time/week, 1-2 times/week, 3-4 times/week, 5–7 times/week), history of hypertension, hypercholesterolemia, cardiovascular disease, and diabetes (each yes, no), unless in the specific subgroup.

**Table 3:** Hazard ratio of clinically diagnosed AAA in association with baseline history of diabetes overall and in subgroups.

Diabetes (yes versus no)	*N*	Basic model^*∗*^	*P*, interaction	Multivariable model^†^	*P*, interaction
All men	471	1.08 (0.78–1.50)		0.79 (0.57–1.11)	
Age			0.59		0.41
<65 years	97	0.75 (0.27–2.05)		0.45 (0.16–1.26)	
≥65 years	374	1.10 (0.78–1.56)		0.86 (0.61–1.22)	
Smoking status			0.59		0.51
Never	120	0.82 (0.38–1.77)		0.65 (0.30–1.42)	
Ever	350	1.08 (0.75–1.55)		0.84 (0.58–1.21)	
Alcohol use			0.73		0.74
Less than daily	262	1.17 (0.78–1.75)		0.86 (0.57–1.30)	
More than daily	202	0.98 (0.55–1.76)		0.72 (0.40–1.30)	
Vigorous exercise			0.36		0.33
Less than once per week	218	1.19 (0.78–1.81)		0.91 (0.60–1.40)	
More than once per week	250	0.85 (0.49–1.46)		0.62 (0.36–1.08)	
Hypertension			0.77		0.90
No	173	1.22 (0.64–2.32)		0.80 (0.42–1.54)	
Yes	297	0.94 (0.64–1.37)		0.81 (0.55–1.18)	
Hypercholesterolemia			0.99		0.98
No	223	1.05 (0.63–1.76)		0.81 (0.48–1.36)	
Yes	242	1.06 (0.70–1.62)		0.80 (0.52–1.24)	
Cardiovascular disease			0.41		0.58
No	362	1.09 (0.73–1.62)		0.83 (0.55–1.25)	
Yes	109	0.80 (0.46–1.41)		0.74 (0.42–1.32)	

^*∗*^Model adjusted for age, race, randomized treatment assignment (aspirin, *β*-carotene, vitamin E, vitamin C, multivitamin).

^†^Model additionally adjusted for body mass index (continuous), smoking status (never, past, current), alcohol use (none, monthly, weekly, daily), vigorous exercise (<1 time/week, 1-2 times/week, 3-4 times/week, 5–7 times/week), and history of hypertension, hypercholesterolemia, cardiovascular disease (each yes, no), unless in the specific subgroup.
